# Negative Pressure Tube Drainage for the Management of Pharyngocutaneous Fistulas in Patients Following Total Laryngectomy

**DOI:** 10.3390/jcm14061854

**Published:** 2025-03-10

**Authors:** Wenwen Diao, Jianlin Fang, Yingying Zhu, Shuting Yu, Xingming Chen, Xiaoli Zhu

**Affiliations:** 1 Department of Otolaryngology-Head and Neck Surgery, Peking Union Medical College Hospital, Peking Union Medical College and Chinese Academy of Medical Sciences, Beijing 100730, China; diaowenwen1@pumch.cn (W.D.); zhuyingying10690@pumch.cn (Y.Z.); yushuting@pumch.cn (S.Y.); chenxm@pumch.cn (X.C.); 2 Department of Otolaryngology-Head and Neck Surgery, Zhongshan Hospital of Xiamen University, School of Medicine, Xiamen University, Xiamen 361005, China; fangjianlin@xmzsh.com

**Keywords:** head and neck cancer, laryngocarcinoma, total laryngectomy, pharyngocutaneous fistula, non-surgical strategy, minimally invasive method

## Abstract

**Background**: The treatment of a pharyngocutaneous fistula (PCF) is typically a lengthy and arduous process, often causing significant pain for patients. This study aims to introduce a new method of treating PCF, negative pressure tube drainage (NPTD). **Methods**: A retrospective study was conducted on 43 patients who developed PCF after laryngectomy. Of these, 20 patients received NPTD, while the remaining 23 were treated with open wound dressing change (OWC). Clinical indicators were compared between the two groups. **Results**: The NPTD group showed significant reductions in hospital stay, duration of low albumin/hemoglobulin, and postoperative medical costs compared to the OWC group. Between the two groups, there was no significant difference in the time from fistula formation to wound closure. The mean healing time was 20.57 days in the OWC group and 22.15 days in the NPTD group, respectively, which did not delay adjuvant therapies. **Conclusions**: NPTD therapy is a minimally invasive and effective treatment option for pharyngocutaneous fistulas.

## 1. Introduction

A pharyngocutaneous fistula (PCF) is one of the most common complications after primary and salvage total laryngectomy (TL), with incidence rates ranging from 3% to 50% [1,2]. The incidence increases up to 76% after high-voltage irradiation [2]. When PCF is diagnosed, the traditional treatment is opening the wound and changing the dressings until it heals. Open wound dressing change (OWC) usually causes a high degree of pain, low serum albumin, an extended hospital stay, a greater nursing workload, and possible surgical approaches to close the wound. Some non-surgical strategies have been reported including managing salivary bypass tubes in total laryngectomy, negative-pressure or vacuum-assisted wound therapy, hyperbaric oxygen therapy, or botulinum toxin injection [3,4,5,6,7,8]. Vacuum-assisted wound therapy (VAT) is an excellent treatment for PCF, having satisfactory results. However, the VAT sponge needs to be changed every 2–5 days, causing varying degrees of pain for the patients [6,7,8]. Additionally, vacuum devices are too expensive for poorer patients to afford. Therefore, we designed a simple and affordable non-surgical method for treating small to medium PCFs.

Negative pressure tube drainage (NPTD) is a minimally invasive technique that allows drainage without surgery or large incisions. In this study, we retrospectively reviewed the results of using NPTD for the treatment of PCFs and compared the outcomes with traditional OWC treatment, including the average length of hospital stay, postoperative medical cost, duration of low albumin after fistula identification, and healing time in a single institution.

## 2. Patients and Methods

### 2.1. Patients

All 725 total laryngectomy (TL) cases (in our hospital) from January 2009 to December 2021 were retrospectively reviewed. Data from 43 patients who developed PCF after TL in this period were collected. The patients who had major necrosis after flap reconstruction were excluded in this study. In all cases, the surgical decision was made by the head and neck multidisciplinary treatment team. Among them, all 23 patients with PCFs identified between January 2009 and December 2016 were treated with OWC, while all 20 patients diagnosed between January 2017 and December 2021 were treated with NPTD. Patient medical data were collected and compared, including age, gender, prior radiation or chemoradiation before surgery, albumin/hemoglobin levels before the surgery and after fistula diagnosis, comorbidities (e.g., diabetes mellitus), tumor stage and clinical staging (AJCC TNM 8th edition), status of margins, length of hospital stay, healing time, and postoperative medical costs. The Ethics Committee of our hospital approved this study.

### 2.2. Surgery

**Figure 1:**
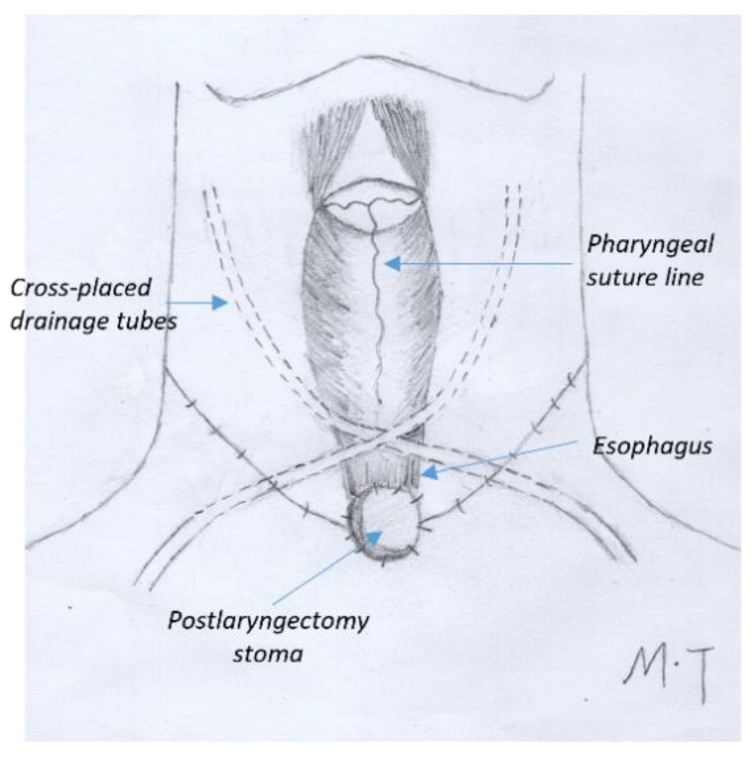
Graphical illustration of cross drainage after total laryngectomy.

Prophylactic antibiotic therapy (2 g intravenous amoxicillin/clavulanate or 1 g cephalosporin plus metronidazole) was administered 1 h before incision. The surgery was performed by two surgical teams. The closure was performed primarily using Vicryl 3-0 sutures or a linear stapler according to the location of the neoplasm [9]. For significant defects with non-adequate remaining tissue conditions, a pectoralis major pedicled flap (PMPF) or a supraclavicular artery island flap (SCAIP) was used according to the surgeon’s intraoperative evaluation of the extent of the defect and the viability of the remaining tissues. Bilateral elective neck dissection was carried out in all cases. Two drainage tubes were placed across each other in the neck (Figure 1). An additional drainage tube was placed in the donor area if a flap was used. Enteral feeding through a nasogastric tube was started after the resumption of peristalsis.

### 2.3. PCF Treatments and Patient Categorization

Patients with PCFs identified before 2017 were treated with OWC, while patients diagnosed with PCFs from 2017–2021 were treated with NPTD. All the patients were discharged from the hospital when they no longer required daily nursing care. Drains were removed 5 to 7 days postoperatively in both groups when the patients had a normal temperature, minimal drainage, and no skin swelling or redness in the neck.

Between 2009 and 2016, wounds were opened in hospital for daily dressing changes when PCF was suspected (either before or after drains were removed). Patients were discharged from the hospital when the pharyngeal fistula had minimal exudate that did not require daily dressing changes. Patients with minimal openings received weekly wound care instructions in the outpatient clinic until the fistulas were completely closed.

**Figure 2:**
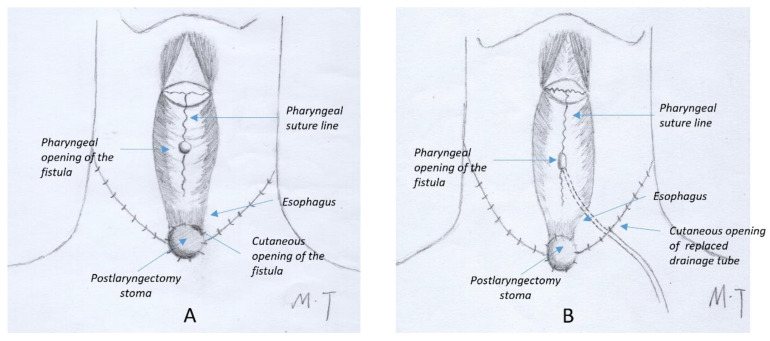
Graphical illustration of a PCF after the drainage tube is removed. (**A**) The pharyngeal opening of the fistula is typically localized around the T-suture of the pharyngeal mucosa. The cutaneous opening of the fistula is typically localized near the tracheostomy stoma. (**B**) Graphical illustration of the new drainage tube placement avoiding proximity to the stoma.

**Figure 3:**
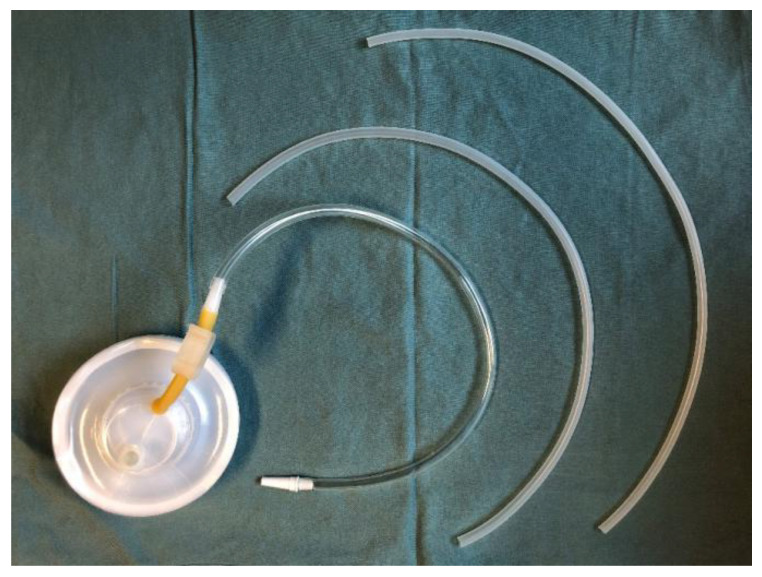
Drainage box and different sizes of drainage tubes.

From January 2017, if PCF was suspected prior to drain removal (due to high temperature, cloudy drainage, or swollen skin or redness in the neck), the drains were left in place and amylase levels in the drainage were tested daily. The tubes were removed 10–14 days after surgery for patients with consistently negative amylase tests. If a patient was diagnosed with a PCF after the drain amylase tested > 4000 IU/L, both tubes were retained in the neck. In these cases, the drainage volume of one tube decreased to a minimum within 1 week of the PCF diagnosis. Therefore, only one tube was necessary to drain saliva leakage in all cases, and the other tube was removed. The patients were discharged with a single drainage tube. Follow-up visits to the outpatient clinic were scheduled on a weekly basis until oral feeding was achieved without any signs of leakage. Subsequently, the tube was removed, and the wound healed naturally within 3 days. For patients diagnosed with PCF after drainage tube removal, the cutaneous openings were usually located near to the laryngectomy stoma (Figure 2A). A silicone drain was placed along the sinus tract from the skin opening. It is preferable to place the drain as deep as possible within the sinus tract, whilst ensuring that the distal end does not exceed the pharyngeal fistula, in order to ensure the maintenance of negative pressure within the sinus tract. In the present study, the sinus tract was meticulously explored prior to the insertion of the drain, and the skin opening was enlarged as necessary to ensure adequate clearance and observation of the sinus tract, thereby minimizing complications. The drain was fixed to the lateral side of the neck, thus ensuring that it was as far away as possible from the tracheostomy opening. It was then sutured to close the skin opening on the medial side of the neck (Figure 2B). After several days of continuous negative pressure suction, the cutaneous opening healed spontaneously. Different sizes of tubes were utilized depending on the extent of fistulas. A box with negative pressure of approximately −120 mmHg was connected to the tube (Figure 3).

### 2.4. Statistical Analysis

SPSS software (SPSS for Windows version 25.0; IBM Corporation, Armonk, NY, USA) was used to analyze the data. A descriptive analysis of all of the clinical and histological parameters was performed. Continuous variables were analyzed using Student’s t-test, and categorical variables were analyzed using a chi-squared test. A multivariate linear regression was used to study the influence of different factors. A *p*-value of <0.05 was considered significant.

## 3. Results

A total of 725 patients underwent TL from 2009 to 2021. Among them, 344 cases were performed from 2009 to 2016, and 381 cases were performed between 2017 and 2021. The PCF incidences were 6.7% (23/344) and 5.2% (20/381), respectively. The characteristics of patients with PCF are listed in Table 1. There were no significant differences in the average age, sex, tobacco and alcohol consumption, comorbidities, TN stages and clinical staging, and prior radiation or chemoradiation treatment between the NPTD and OWC groups. The surgical margins were negative in all cases in both groups.

The length of hospital stay was significantly reduced to 15 days in the NPTD group from 28 days in the OWC group. The duration of low albumin/hemoglobulin and the postoperative medical cost were also significantly reduced compared to the OWC group. No significant changes in duration from fistula onset to wound closure were observed between the groups (Table 2).

The results of the multivariate linear regression analysis showed that treatment modality significantly affected the length of hospital stay, the duration of low albumin/hemoglobulin, and the postoperative medical cost, while age, diabetes mellitus, and comorbidities had no significant effect on these indicators (Table 3).

## 4. Discussion

The management of pharyngocutaneous fistulas includes the identification of risk factors preoperatively. The incidence of PCF is significantly increased in patients with COPD, CAD, diabetes mellitus, previous radiation or chemoradiation, preoperative low albumin or hemoglobin, salvage surgery, extended total laryngectomy (including pharyngectomy), positive surgical margins, postoperative hypoproteinemia, and early oral feeding [10,11,12,13]. Blood glucose instability, anemia, and malnutrition should be aggressively corrected throughout the perioperative period [14,15]. In patients having high risk factors, early oral feeding needs to be avoided [16]. Crosettis et al. reported the results of a “fistula-zero project” after TL to reduce PCF rates by using a watertight horizontal pharyngeal closure, a reinforcing flap for post-radiation patients, and the use of salivary bypass tubes [17]. In our study, low albumin/hemoglobin and unstable blood glucose in diabetes patients were corrected during the perioperative period. A reinforced suture outside the anastomosis was performed in salvage laryngectomy. The drainage tubes were crossed in the neck to prevent liquid accumulation or hematoma in the central part of the neck around the anastomosis.

Besides prevention, the early diagnosis of PCF is crucial for shortening wound healing time [18]. PCF is usually diagnosed 7 to 11 days after surgery [16]. In our experience, patients with continuous low-grade fever, high white blood cell counts with a high proportion of neutrophils, and red or swollen cervical skin postoperatively had an increased risk of developing PCF. These risk factors should be normalized before drainage is removed. Alternatively, the neck drains should be left in place. For patients who did not have such risk factors in the postoperative period and were diagnosed with PCF after drain removal, a new tube was put in place following the procedure outlined in this paper.

Different methods have been reported to treat the fistula after a PCF diagnosis. Surgical closure of the wound was considered to be an effective method [4,5]. In surgical management, debridement with a fistula tract suture rarely achieves complete healing. In most cases, a pedicled flap (e.g., pectoralis major myocutaneous flap) or free flap reconstruction are required for definitive repair. Notably, the healing time of surgical treatment is much longer than non-surgical wound care [19]. Various non-surgical treatment modalities for PCF have been reported in the literature including hyperbaric oxygen therapy, alginate dressings with intense draining properties, botulinum toxin injection, or negative pressure (or vacuum-assisted) therapy [6,7,20,21,22]. Negative pressure therapy for PCF is widely used with various devices [6,8,23]. Most vacuum-assisted devices consist of a continuous system with negative pressure and a sponge or dressing covering the wound. The negative pressure ranges from −100 to −125 mmHg, and the sponge or dressing needs to be changed every 2 to 5 days. These devices are suitable for open wounds with a cavity and have proven efficacy in reducing the healing time. However, it was observed that the fistula typically showed a small amount of saliva leakage during the initial identification phase, as well as small gaps around the fistula. In our experience, no obvious infection or necrosis was observed when PCF was diagnosed in cases with prolonged use of drainage tubes. We hypothesized that this could be a new method to treat minor PCFs. In such cases, the tube could remain in place with a continuous negative pressure of −120 mmHg, which is effective and adequate for drainage and wound healing. Continuous negative pressure suction from the device installed on the wall of the ward was utilized in situations of air leakage, and all patients developed air-tight drainage through the tube after 5 days. For PCF cases diagnosed subsequent to tube removal, the wounds tended to be in close proximity to the laryngectomy stoma. We incised the wound to remove the necrotic tissue and exudate and then inserted a drainage tube, avoiding the laryngectomy stoma. The initial injuries were sutured and healed without intervention within 3 to 5 days. As the PCF had healed with the replacement drainage tube in place, the patients were included in our study group.

After the patients were discharged from the hospital, no dressing change or special nursing care was needed. The volume of drainage decreased significantly in most patients 2 weeks after discharge, and approximately 1 week later, the patients attempted oral feeding and the tube was removed after confirmation of no pharyngeal fistula. We postulated that constant negative pressure could effectively drain secretions, promoting optimal surgical wound healing and rapid granulation development around the PCF. Upon removing the drainage tubes at the clinic, we discovered that the side holes of the tubes were densely populated with granulation. These observations confirm our hypothesis.

Compared to conventional open wound care, the novel technique eliminates the discomfort experienced during dressing changes and shortens hospital stays, thereby reducing the average cost of treatment for PCF. Another benefit of NPTD was improved patient nutrition. This could be attributed to the alleviation of pain and decreased protein exudation or nutrient loss through closed wound care. Finally, a crucial aspect of our novel approach is that the mean recovery period aligns with that of the conventional method. Therefore, there was no delay in the initiation of adjuvant treatments.

Given the possibility that age, diabetes, other comorbidities, and treatment modalities could have impacted the clinical indicators observed, we conducted a multivariate linear regression analysis to explore the influencers of the duration of hospitalization, low levels of albumin/hemoglobulin, and postoperative medical expenses in this patient cohort. After excluding the effects of multiple factors, the treatment method remained a significant factor influencing the duration of hospitalization, malnourishment, and postoperative healthcare costs. Therefore, our results have shown that NPTD treatment is a more manageable and cost-effective option for PCF compared to OWC treatment.

The current study presented a minimally invasive method using a drainage tube to treat PCFs after total laryngectomy. However, it has some limitations. Firstly, the sample size was relatively small due to the low rate of PCF. Furthermore, this is a retrospective study. As we noted the benefits of NPTD in clinical work, the patients with PCFs after 2017 were no longer treated with OWC. Consequently, the two groups of patients were treated during different time periods, potentially introducing more confounding variables into this study. Additionally, this study was based on the experiences of a single center, which may limit its applicability to other surgeons and hospitals.

## 5. Conclusions

The management of pharyngocutaneous fistula can be a time-consuming and labor-intensive process. Using the suction method is no worse than using a wound dressing and does not appear to cause any harm. The negative pressure tube drainage is an easy-to-perform and safe procedure especially in cases where there is no apparent necrosis or abscess around the fistula. So we believe that the minimally invasive technique of negative pressure tube drainage is a successful method for the closure of PCFs. Identification of risk factors and placement of cross-drainage tubes during laryngectomy may prevent the need for aggressive interventions. Future prospective studies with multicenter cohorts and larger sample sizes are necessary to verify these findings.

## Figures and Tables

**Table 1:** Patient characteristics.

Clinicopathological Feature	Total (N)	OWC Group	NPTD Group	*p*-Value
All cases	43	23	20	
Age (y), mean ± SD	60.30 ± 7.41	59.35 ± 5.71	61.40 ± 9.01	0.371
Sex				0.468
Male	40 (93.02%)	22 (95.65%)	18 (90.00%)	
Female	3 (6.98%)	1 (4.35%)	2 (10.00%)	
Tobacco use				0.883
Yes	39 (90.70%)	21 (91.30%)	18 (90.00%)	
No	4 (9.30%)	2 (8.70%)	2 (10.00%)	
Alcohol use				0.669
Yes	23 (58.49%)	13 (56.52%)	10 (50.00%)	
No	20 (46.51%)	10 (43.48%)	10 (50.00%)	
Diabetes				0.536
Yes	11 (25.58%)	5 (21.74%)	6 (30.00%)	
No	32 (74.42%)	18 (78.26%)	14 (70.00%)	
Low albumin/hemoglobin Before surgery				0.538
Yes	36 (83.72%)	20 (86.96%)	16 (80.00%)	
No	7 (16.28%)	3 (13.04%)	4 (20.00%)	
Comorbidities				0.069
Present	26 (60.47%)	12 (52.17%)	15 (75.00%)	
Absent	17 (39.53%)	11 (47.83%)	5 (25.00%)	
TN stage *				0.373
T_3_N_0_/N+	9 (20.93%)	6 (26.09%)	3 (15.00%)	
T_4_N_0_/N+	34 (79.07%)	17 (73.91%)	17 (85.00%)	
Clinical stage *				0.345
Ⅲ	1 (2.33%)	1 (4.35%)	0 (0.00%)	
Ⅳ	42 (97.67%)	22 (95.65%)	20 (100.00%)	
Prior irradiation/CRT				0.149
Present	7 (16.28%)	2 (8.70%)	5 (25.00%)	
Absent	36 (83.72%)	21 (91.30%)	15 (75.00%)	
Flap reconstruction				0.520
Present	5 (11.63%)	2 (8.70%)	3 (15.00%)	
Absent	38 (88.37%)	21 (91.30%)	17 (85.00%)	

OWC: open wound dressing change; NPTD: negative pressure tube drainage; * staging based on AJCC TNM 8th edition; CRT: chemoradiation.

**Table 2:** Comparison of duration of hospital stay, low albumin/hemoglobin, fistula closure, and total medical cost between the two groups.

	OWC Group	NPTD Group	*p*-Value
Length of hospital stay (days, mean ± SD)	28.70 ± 6.54	15.10 ± 2.32	0.000
Duration of low albumin/hemoglobin (days, mean ± SD)	4.70 ± 1.94	2.90 ± 1.68	0.003
Duration from fistula onset to its closure (days, mean ± SD)	20.57 ± 6.10	22.15 ± 4.86	0.264
Postoperative medical cost (1000 RMB, mean ± SD)	30.25 ± 4.73	24.45 ± 3.35	0.000

OWC: open wound dressing change; NPTD: negative pressure tube drainage.

**Table 3:** Influence of different factors on the length of hospital stay, the duration of low albumin/hemoglobulin, and the total medical cost.

	Length of Hospital Stay	Duration of Low Albumin/Hemoglobin	Postoperative Medical Cost
	(*p*-Value)	(*p*-Value)	(*p*-Value)
PCF treatments	0.000	0.000	0.000
Age	0.774	0.092	0.430
Diabetes	0.066	0.079	0.454
Comorbidities	0.402	0.766	0.279

PCF: pharyngocutaneous fistula.

## Data Availability

The original contributions presented in this study are included in the article. Further inquiries can be directed to the corresponding author(s).
